# Multimodal AI mapping with GigaTIME reveals spatial immune signaling states in the tumor microenvironment

**DOI:** 10.7150/ijbs.130729

**Published:** 2026-03-30

**Authors:** Hakjin Kim, Seon-Kyu Kim, Jinhyuk Lee, Sung-Hwan Moon, Sun-Uk Kim, Taeho Kwon

**Affiliations:** 1 Quantum AI Bio Research Laboratory (KJQI-JQL), In Quantio, Gene on Biotech, Daejeon 35229, Republic of Korea.; 2 Advanced Bioconvergence Department, Department of Bioscience, Department of Bioinformatics, KRIBB School, Korea National University of Science and Technology (UST), Daejeon 34113, Republic of Korea.; 3 AI-Bio Solution Team, Genomic Medicine Research Center, Korea Research Institute of Bioscience and Biotechnology (KRIBB), Daejeon 34141, Republic of Korea.; 4 Bio-design Editing Research Center, Korea Research Institute of Bioscience and Biotechnology (KRIBB), Daejeon 34141, Republic of Korea.; 5 Department of Animal Science and Technology, College of Biotechnology, Chung-Ang University, Anseong 17546, Republic of Korea.; 6 Futuristic Animal Resource and Research Center, Korea Research Institute of Bioscience and Biotechnology (KRIBB), Cheongju, Chungbuk 28116, Republic of Korea.

Understanding how immune signaling states are spatially organized within tumors is central to deciphering tumor-immune interactions and therapeutic responses. Recent advances in artificial intelligence (AI) have begun to expand the analytical potential of routine pathology, enabling large-scale investigation of the tumor immune microenvironment. In a recent study published in *Cell*, Valanarasu *et al*. introduced GigaTIME, a multimodal artificial intelligence (AI) framework that translates routine hematoxylin and eosin (H&E) stained pathology slides into virtual multiplex immunofluorescence (mIF) images, enabling population-scale modeling of the tumor immune microenvironment (TIME) 1. GigaTIME bridges tissue morphology with inferred protein activation states, enabling systematic analysis of immune checkpoint-related signaling patterns, including PD-1 and PD-L1, across more than 14,000 patients representing 24 distinct cancer types (Figure 1).

Although multiplex immunofluorescence has emerged as a powerful approach for interrogating spatial immune signaling within tumors, however, its application in large-scale clinical discovery remains constrained due to the high cost, technical complexity, and limited throughput 2. To overcome these barriers, Valanarasu *et al*. proposes a cross-modal learning strategy, in which paired H&E and mIF datasets are used to train a translator, which in turns, is able to infer protein activations directly from histological features 1. The training dataset comprises approximately 40 million cells across 21 TIME-associated protein markers, allowing the model to capture a broad spectrum of immune, tumor, and stromal states. This multimodal AI framework generates a large and diverse virtual population containing spatially resolved activation maps for immune checkpoints, immune cell markers, proliferation, and apoptosis, allowing for previously infeasible analyses at this scale.

Importantly, the conceptual novelty of GigaTIME does not lie in predicting protein markers from pathology images per se, but in enabling population-scale, spatially resolved modeling of immune signaling states. Across the virtual cohort, immune checkpoint-associated activation patterns display robust and statistically significant associations with established clinical biomarkers and pathological features, thereby supporting the relevance of AI-inferred immune signaling states 1, 3. Notably, virtual checkpoint activation, exemplified by PD-L1, correlates positively with clinically reported PD-L1 immunohistochemistry scores, indicating concordance between AI-generated virtual mIF signals and standard diagnostic assays used in clinical practice 1. This agreement supports the biological validity of morphology-derived virtual protein activation in capturing clinically relevant immune checkpoint information.

In parallel, increased checkpoint-associated activation is negatively associated with multiple immune infiltration (including CD3-, CD8-, and CD20-positive lymphocyte), proliferation (Ki67 and PHH3), and apoptosis (caspase-3) markers 1. These inverse relationships delineate immune-suppressive tumor contexts characterized by reduced lymphocyte presence and diminished proliferative and apoptotic activity, consistent with previously described immuneexcluded or immune-dysregulated microenvironments 4. Importantly, these associations emerge at the population level across diverse tumor types, underscoring the capacity of virtual mIF for capturing broad immune signaling trends from routine pathology.

Rather than relying on single-channel assessment, GigaTIME allows for the systematic combinatorial analysis of protein activation patterns, a key methodological advantage of the multimodal AI framework 1. Joint evaluation of protein channel pairs reveals associations that are not apparent when markers are analyzed in isolation 1. In particular, pairwise integration of checkpoint-related activation with cell fate markers (e.g., caspase-3) exhibits significant and consistent associations with clinical biomarkers, rather than marker analyzed alone. This observation illustrates how immune checkpoint-related signals could be contextualized along with complementary cell state indicators, providing a more nuanced immune landscape characterization within tumors. Such combinatorial analyses highlight the limitations of single-marker readouts and demonstrate the added interpretive value gained through integrated signaling assessment 5.

Importantly, checkpoint-associated activation patterns exhibit substantial heterogeneity across cancer types and molecular subtypes, thereby emphasizing the context-dependent immune signaling organization within the tumor immune microenvironment 1. For example, despite arising from the same organ, lung adeno- and squamous cell carcinomas exhibit distinct relationships between checkpoint-related activation, immune infiltration markers, and pathological staging. These subtype-specific patterns indicate that immune checkpoint signaling is shaped by tumor-intrinsic and microenvironmental factors unique to each cancer context. Such variability provides a plausible framework for understanding the heterogeneous clinical responses observed with immune checkpoint inhibitors, underscoring the need for context-aware immune profiling strategies.

This study highlights the broader potential of multimodal artificial intelligence to reshape how immune signaling is studied using routine clinical pathology. By converting standard H&E-stained slides into scalable, spatially resolved representations of protein activation, GigaTIME illustrates how histopathology could serve as a basis for population-level signal transduction analysis rather than a purely descriptive diagnostic tool. The hereby-described cross-modal translation strategy is not inherently restricted to PD-1/PD-L1 signaling, suggesting its applicability to other immune and non-immune pathways, disease settings, and therapeutic targets. As digital pathology datasets continue to grow, multimodal AI frameworks such as GigaTIME may enable the systematic reuse of existing clinical material for biomarker discovery, comparative signaling analysis, and translational research, thereby amplifying the impact of AI in precision immunotherapy and beyond, particularly as advanced computational paradigms, such as high-performance computing and emerging quantum computational approaches, continue to evolve.

## Figures and Tables

**Figure 1:**
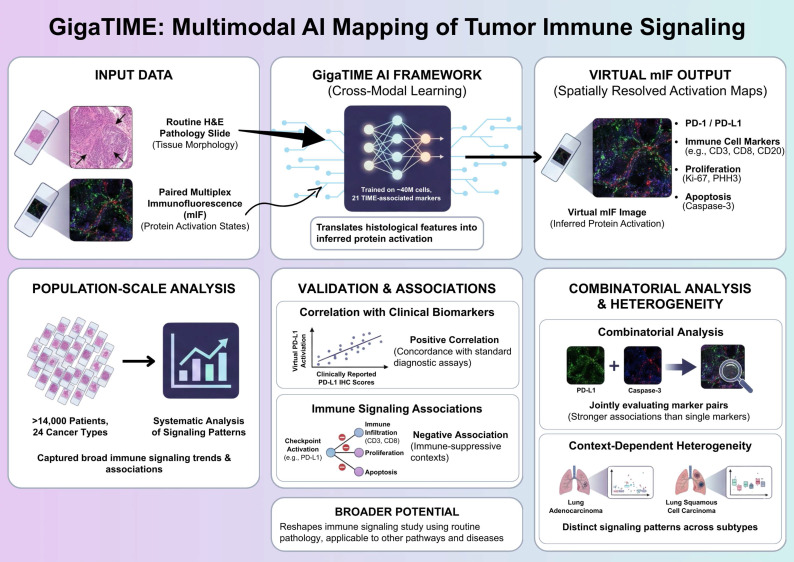
** Multimodal AI-based mapping of spatial immune signaling in the tumor immune microenvironment.** GigaTIME framework maps spatial immune signaling from routine pathology using a multimodal artificial intelligence approach. Paired hematoxylin and eosin (H&E) and multiplex immunofluorescence (mIF) images are used to train a cross-modal model that translates tissue morphology into inferred protein activation states. The trained model generates virtual mIF images with spatially resolved activation maps, capturing immune checkpoint-associated signals, immune cell markers, and proliferation and apoptosis indicators while preserving tissue architecture. GigaTIME application to more than 14,000 patients across 24 cancer types allows for population-scale immune signaling pattern analyses, validation against clinical biomarkers, and combinatorial signaling and context-dependent heterogeneity assessment across tumor types and molecular subtypes. Figure created with TOV studio (https://www.tov.studio/).

## Abbreviations

AI: Artificial intelligence
H&E stain: Hematoxylin and eosin stain
mIF: Multiplex immunofluorescence images
TIME: Tumor immune microenvironment
PD-1: Programmed Death-1
PD-L1: Programmed Death-Ligand 1
